# Reduced abundance of *Fusobacterium* signifies cardiovascular benefits of sodium glucose cotransporter 2 inhibitor in type 2 diabetes: a single arm clinical trial

**DOI:** 10.3389/fphar.2025.1600464

**Published:** 2025-05-08

**Authors:** Shuhui Yang, Jiankun Deng, Xiaoxu Weng, Zhaojie Ma, Nie Lin, Yili Xiao, Rui Zuo, Yufei Hu, Canbin Zheng, Xiaoshan Zeng, Qimao Lin, Kaijian Hou

**Affiliations:** ^1^ Department of Endocrine and Metabolic Diseases, Shantou Central Hospital, Shantou, China; ^2^ School of Public Health, Shantou University, Shantou, China; ^3^ Department of Endocrine and Metabolic Diseases, Longhu People’s Hospital, Shantou, China

**Keywords:** sodium glucose cotransporter 2 inhibitor, type 2 diabetes, cardiovascular disease, gut microbiota, *Fusobacterium*

## Abstract

**Background:**

The sodium glucose cotransporter 2 inhibitor (SGLT2i) dapagliflozin has been demonstrated cardiovascular benefits in patients with type 2 diabetes mellitus (T2DM). However, the underlying mechanism remains poorly understood.

**Methods:**

We conducted an 8-week, single-arm clinical trial, which enrolled 12 patients with inadequate glycemic control on metformin monotherapy. These patients were treated with SGLT2i dapagliflozin (10 mg/day). We assessed changes in clinical parameters pertinent to glucose metabolism and risk factors of cardiovascular disease (CVD), as well as alterations in the gut microbiota using macrogene sequencing.

**Results:**

Improvements were observed in anthropometric parameters, glucose metabolism, blood lipid-related indices, inflammatory markers, and endothelial cell function-related parameters. Concurrently, SGLT2i led to changes in composition and functional pathways of the gut microbiota, manifested as increased abundance of probiotics and decreased abundance of harmful bacteria. Importantly, reduced abundance of *Fusobacterium* was correlated with improvements in various clinical indicators.

**Conclusion:**

SGLT2i represents a superior initial therapeutic option for T2DM patients at risk of CVD. The cardiovascular benefits of SGLT2i may be attributed to shifts in the gut microbiota, particularly the reduced abundance of *Fusobacterium*.

## 1 Introduction

The primary contributor to morbidity and mortality of patients with type 2 diabetes mellitus (T2DM) is cardiovascular diseases (CVDs) (Eckel et al., 2006). Sodium-glucose cotransporter 2 inhibitors (SGLT2i) have been developed as hypoglycemic drugs that target SGLT2, the major glucose transporter in the kidney responsible for about 90 percent of glucose reabsorption from primary urine (Scheen, 2015). Beyond achieving glucose-lowering effects, SGLT2i was reported to reduce major adverse cardiovascular events and mortality in T2DM patients at high risk of CVD or with established CVD (Wanner et al., 2018; Neal et al., 2017; Zinman et al., 2015). The CVD-REAL 2 study suggested that SGLT2 inhibitors, when compared with other glucose-lowering medications, were associated with a reduced cardiovascular risk (Kosiborod et al., 2018). According to Standards of Care in Diabetes 2025 published by American Diabetes Association, sodium glucose cotransporter 2 inhibitor (SGLT2i) was recommended to use for glycemic management and comprehensive cardiovascular risk reduction in adults with T2DM and established or high risk of cardiovascular disease (American Diabetes Association Professional Practice Committee, 2025).

The cardiovascular protective effects conferred by SGLT2i might be attributed to their diuretic properties, weight loss promotion, glycemic control, and blood pressure regulation (Perrone-Filardi et al., 2017; Liu et al., 2021). Nonetheless, the underlying mechanisms responsible for the cardiovascular benefits of SGLT2i remain unclear. Pharmacologically, SGLT2i enhance urinary glucose excretion by targeting the SGLT2 receptor of renal proximal tubule (Tahrani et al., 2013). Meanwhile, these inhibitors might act on SGLT1, which is highly expressed in the gastrointestinal tract, to reduce intestinal glucose uptake (van Bommel et al., 2017; Han et al., 2008). The changed microenvironment may alter microbiota and related metabolites formation (Lehmann and Hornby, 2016). The gut microbiota is a complex and dynamic entity composed of trillions of microorganisms that live in close symbiosis with their host, consisting of different species of bacteria (Eckburg et al., 2005; Almeida et al., 2019). To date, substantial evidences of gut microbiota dysbiosis have been found in T2DM individuals (Zhou et al., 2022). Emerging evidence showed that the gut microbiota was involved in chronic inflammation, metabolic disorders and oxidative in the host, contributing to the progression of T2DM and CVDs (Yang et al., 2021; Wu et al., 2023; Nemet et al., 2020; Wang et al., 2011; Raygan et al., 2018; Yuan et al., 2019). Targeting the gut microbiota was considered as an emerging therapy for T2DM and diabetic cardiovascular diseases (Yang et al., 2021). Animal study has uncovered that SGLT2i improves generalized vascular dysfunction which associated with alterations of gut microbiota in T2DM models (Lee et al., 2018; Yang M. et al., 2020). Moreover, it was reported that the cardiovascular benefits of SGLT2i empagliflozin was associated with changes in the gut microbiota in patients with T2DM (Deng X. et al., 2022).

In this study, we conducted a single-arm clinical trial to investigate the potential association between the cardioprotective effects of SGLT2i and changes in gut microbiota in patients with T2DM.

## 2 Materials and methods

**FIGURE 1:**
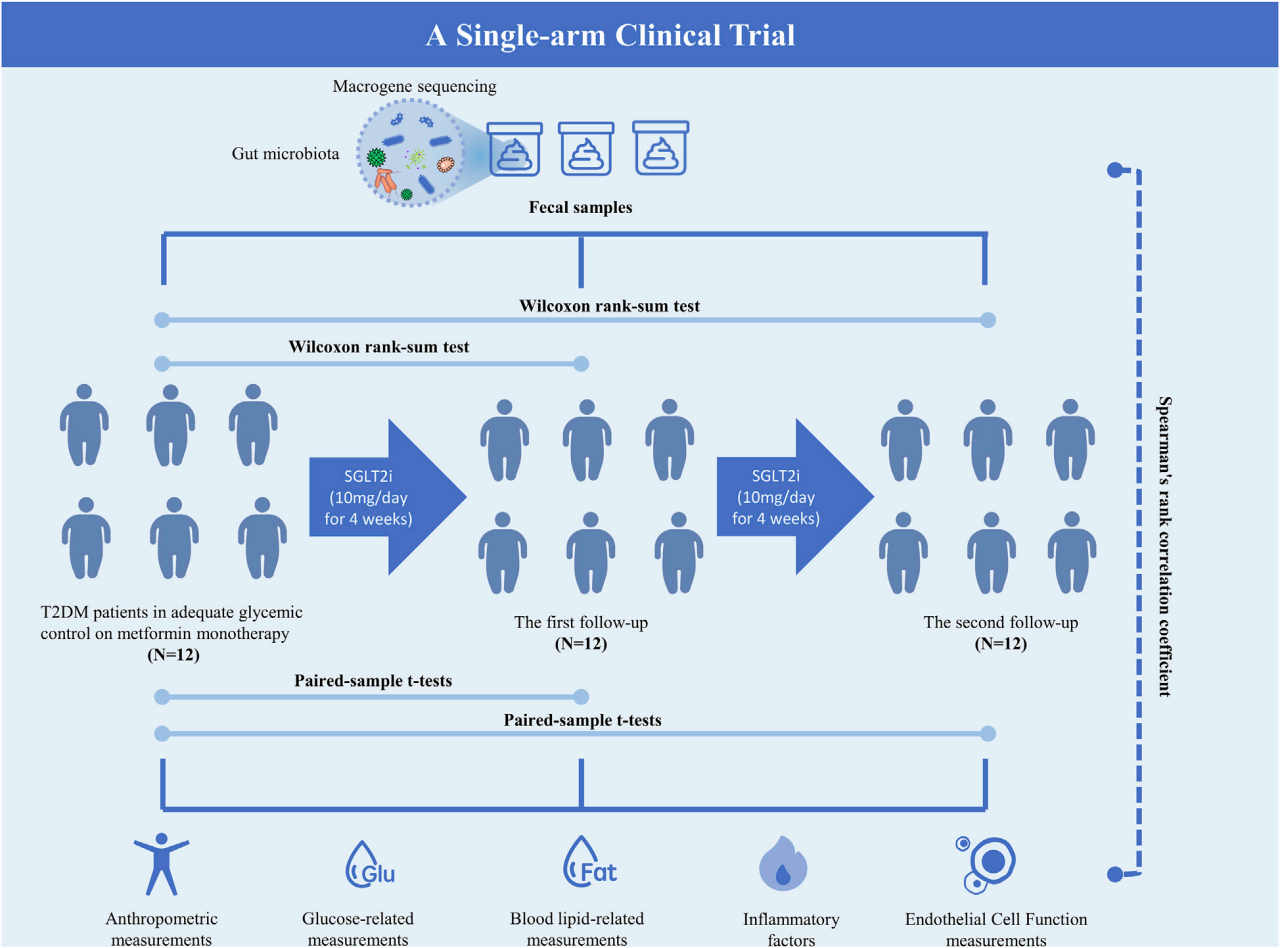
Flowchart of the study.

The flowchart of this study is presented in Figure 1.

### 2.1 Patient recruitment

Subjects included in this study met the following criteria: 1) male and female participants aged between 20 and 75 years; 2) patients diagnosed with T2DM who were on treatment with metformin yet demonstrated inadequate blood glucose control; 3) glycated hemoglobin (HbA1c) levels ranging from 6.3% to 10.5%; and 4) fasting C-peptide (FCP) levels exceeding 1 nmol/L. The diagnosis for T2DM was based on the WHO 1999 criteria: the presence of typical diabetic symptoms along with a random blood glucose level ≥11.1 mmol/L; diabetic symptoms plus a fasting plasma glucose (FPG) level ≥7.0 mmol/L; diabetic symptoms accompanied by a 2-h postprandial blood glucose level ≥11.1 mmol/L; or a confirmed diagnosis of diabetes mellitus following an oral 75 g anhydrous glucose tolerance test (OGTT) with a 2-h post-load glucose (2HPG) level ≥11.1 mmol/L. Diabetic symptoms encompass polydipsia, polyuria, polyphagia, and unexplained weight loss. For individuals without a prior diagnosis of diabetes, a single blood glucose value meeting the diagnostic criteria necessitates confirmation through repeat testing on a subsequent day. Random blood glucose refers to a measurement taken at any time of a day, irrespective of timing of the last meal and caloric intake. Fasting status is defined as the absence of caloric intake for at least 8 h. A total of 12 patients were enrolled. The investigators maintained the records of pre-screened subjects in a subject screening log. The clinical study adhered to the principles outlined in the Declaration of Helsinki and the official Chinese regulations governing clinical research studies. Subjects were only enrolled in the clinical study after providing voluntary informed consents, and patient privacy was diligently maintained and ensured by the investigators.

The Ethics Committee of the SHANTOU Central Hospital approved the study (No. 2020-001). This study was registered with ClinicalTrials.gov (ChiCTR2000029927). The study was conducted in compliance with the Declaration of Helsinki.

### 2.2 Exclusion criteria

Subjects were excluded from this study if any of the following criteria were met: 1) Patients diagnosed with diabetes mellitus other than T2DM; 2) Individuals with severe combined diabetic complications, including diabetic ketoacidosis, hyperosmolar hyperglycemic syndrome, or lactic acidosis; 3) Patients with clinically significant hepatobiliary diseases, such as chronic active hepatitis and/or severe hepatic insufficiency, cirrhosis, glutamic aminotransferase (ALT) or glutamic oxalacetic aminotransferase (AST) levels exceeding three times the upper limit of normal (150 U/L), or serum total bilirubin (TB) levels greater than 34.2 μmol/L (>2 mg/dL); 4) Patients with a history of renal disease or features indicative of renal impairment, including unstable or rapidly progressive renal disease, moderate/severe renal impairment or end-stage renal disease, estimated glomerular filtration rate (eGFR) < 60 mL/min/1.73 m^2^, serum creatinine (Cr) ≥ 133 μmol/L (≥1.50 mg/dL) in male subjects, and serum Cr ≥ 124 μmol/L (>1.40 mg/dL) in female subjects; 5) Patients with any of the following cardiovascular conditions: myocardial infarction, cerebral infarction, cardiac surgery or revascularization procedures (coronary artery bypass grafting/percutaneous transluminal coronary angioplasty), unstable angina, congestive heart failure (New York Heart Association class III or IV), transient ischemic attack, or significant cerebrovascular disease within the past 12 weeks; 6) Individuals with a history of gastrointestinal disease or surgery, including intestinal obstruction, intestinal ulcer, bariatric surgery, girdle surgery, gastrointestinal anastomosis, or bowel resection; 7) Pregnant or breastfeeding women; 8) Patients with a urinary tract infection within the last 2 weeks; 9) Subjects deemed by the investigator to be unlikely to comply with the study protocol, or patients with serious physical or psychological illnesses that may impact the effectiveness or safety of the study.

### 2.3 Interventions

The 12 patients who had poorly controlled T2DM despite treatment with metformin alone were treated with dapagliflozin at a dosage of 10 mg daily. All participants met the inclusion criteria and did not meet any exclusion criteria.

### 2.4 Data collection

Follow-up visits were conducted at the hospital at baseline (week 0), week 4, and week 8. During monthly visit, anthropometric assessments including Body Mass Index (BMI), waist circumference (WC) were performed. Clinical samples collected at baseline and at monthly visits as follow: fecal samples, fasting blood glucose (FBG), 2-hour postprandial glucose (2HPG), fasting insulin (FINS), 2-hour postprandial insulin (2INS), fasting C-peptide (FCP), 2-hour postprandial C-peptide (2HCP), Homeostasis model assessment of insulin resistance (HOMA-IR), HOMA-β cell function index (HOMA-β), glycated hemoglobin levels (HbA1c), interleukin-6 (IL-6), interleukin-8 (IL-8), interleukin-18 (IL-18), interleukin-37 (IL-37), monocyte chemoattractant protein-1 (MCP-1), and tumor necrosis factor-alpha (TNF-α), soluble intercellular adhesion molecule-1 (sICAM-1), vascular cell adhesion molecule-1 (VCAM-1), P-selectin, nitric oxide (NO), prostacyclin (PGI2), and endothelin-1 (ET-1), tissue factor (TF), tissue-type fibrinogen activator (t-PA), von Willebrand factor (vWF), and plasminogen activator inhibitor-1 (PAI-1). Besides, blood lipid-related measurements, renal and liver function were measured.

### 2.5 Macrogene sequencing of intestinal microbiota

Fecal samples were obtained from patients with T2DM on the day of their clinical examination and preserved in the microbiota stabilizer EffcGut until subsequent DNA extraction (Yang L. et al., 2020). Genomic DNA was isolated from these fecal samples utilizing the QIAamp Fast DNA Stool Mini Kit (Qiagen, CA, United States). The extracted DNA was then fragmented to an insert size of 400 base pairs for library preparation and sequenced using the Illumina NovaSeq platform with paired-end 150 reagents. Raw sequencing reads were trimmed to remove sequencing adapters, low-quality reads, and sequences aligning with the human genome (based on the hg18 reference). Microbial gene profiles and KEGG orthologous groups (KOs) were generated by aligning the high-quality reads to a reference gene catalog (Li et al., 2014). Taxonomic composition at the genus, species, and strain levels was determined using MetaPhlAn2 (Segata et al., 2012). The structural composition and functional predictions of bacterial diversity were compared among the Week0, Week4, and Week8 groups treated with SGLT2i. Furthermore, metagenomic sequencing was conducted on selected samples, followed by sequence assembly and functional annotation to investigate the role of microbial communities.

### 2.6 Statistical analysis

For datasets exhibiting normal distributions, paired-sample t-tests were employed to assess the differences of groups. For datasets exhibiting non-normal distributions and microbial characteristics, the Wilcoxon rank-sum test was utilized for intergroup comparisons. Spearman’s rank correlation coefficient was applied to determine the correlation between clinical data and microbial taxa. Data visualization was conducted using the R software (Mair et al., 2015), primarily utilizing the packages reshape2 (Zhang, 2016), ggplot2, ggsignif, ape (Paradis et al., 2004), and gridExtra.

## 3 Results

### 3.1 SGLT2i improves glucose and lipid metabolism and CVD-related risks in T2DM patients

In this study, a total of clinical data from 12 patients with T2DM were analyzed at baseline (W0), week 4 (W4), and week 8 (W8) treatment with SGLT2i. Following a 4-week intervention period, improvements were observed in BMI, WC, 2HPG, FCP, LDLC, IL-6, and cardiovascular endothelium-related indicators, including P-selectin, PGI2, ET-1, TF, t-PA, vWF, and PAI-1. Furthermore, enhancements in BMI, WC, 2HPG, LDLC, HOMA-IR and the cardiovascular endothelium-related indicators were observed at week 8 (Table 1). Except for CVD common risk factors including obesity, hyperglycemia, dyslipidemia (Marx et al., 2023), SGLT2i improved endothelial dysfunction, which was considered to be an independent predictor of CVD in patients with T2DM (de Jager et al., 2006).

**TABLE 1:** Clinical outcomes after SGLT2i intervention.

Characteristic	W0	W4	W8	Difference between W4 and W8 in change from baseline (*95% CI*)	
				W0 to W4	W0 to W8
N	12	12	12		
Male [n/%)]	8 (66)	8 (66)	8 (66)		
Age(y)	62 ± 10.6	62 ± 10.6	62 ± 10.6		
Anthropometric measurements					
BMI (kg/m^2^)	21.6 ± 4.8	21.3 ± 4.6	21.0 ± 4.4	0.3 (0.0, 0.5)*	0.6 (0.1, 1.0)*
WC (cm)	79.7 ± 8.0	79.1 ± 7.8	78.4 ± 7.5	0.6 (0.1, 1.1)*	1.3 (0.4, 2.2)*
WHR	0.85 ± 0.12	0.84 ± 0.1	0.82 ± 0.09	0.02 (0.00, 0.04)	0.03 (0.00, 0.06)*
Glucose-related measurements					
FBG (mmol/L)	10.5 ± 4.4	8.7 ± 2.4	7.7 ± 1.9	1.8 (-0.3, 3.9)	2.8 (0.3, 5.4)*
HbA1c (%)	8.8 ± 1.8	8.5 ± 1.9	8.1 ± 2.0	0.2 (-0.6, 1.0)	0.7 (-0.2, 1.5)
2HPG (mmol/L)	17.1 ± 4.1	11.6 ± 3.2	10.8 ± 3.6	5.5 (2.3, 9.2)**	6.3 (2.6 10.0)**
FCP (ng/mL)	2.6 ± 2.5	2.8 ± 2.4	2.9 ± 2.3	−0.3 (-0.5, −0.0)*	−0.4 (-0.9 0.2)
2HCP (ng/mL)	4.8 ± 2.9	5.7 ± 3.3	5.8 ± 3.7	−0.9 (-1.9, −0.2)	−1.0 (-2.0, 0.1)
FINS (μIU/mL)	11.3 (6.4, 23.1)	11.6 (7.1, 17.4)	9.9 (8.3, 16.5)	4.0 (-0.1, 8.0)	6.0 (-0.7, 12.6)
2INS (μIU/mL)	35.9 (25.9, 55.9)	28.8 (14.6, 50.5)	30.2 (11.0, 45.7)	15.6 (-0.4, 31.6)	27.6 (-4.5, 59.8)
HOMA-IR	8.5 ± 7.9	5.8 ± 4.7	4.2 ± 2.0	2.7 (-0.7, 6.2)	4.3 (0.2, 8.4)*
HOMA-β	36.1 (26.3,160.7)	54.8 (26.8, 93.2)	55.1 (31.1,111.7)	265.5 (-332.6,863.5)	249.4 (-351.9,850.8)
Blood lipid-related measurements					
TC (mmol/L)	5.1 ± 1.3	5.1 ± 1.1	5.2 ± 1.5	0.0 (-0.8, 0.8)	−0.1 (0.9, 0.6)
TG (mmol/L)	1.4 (1.3, 2.2)	1.2 (0.9, 3.1)	1.0 (0.7, 2.3)	0.5 (-0.8, 1.8)	0.5 (-0.8, 1.8)
HDLC (mmol/L)	1.3 ± 0.2	1.3 ± 0.3	1.3 ± 0.3	−0.1 (-0.2, 0.1)	0.0 (-0.1, 0.1)
LDLC (mmol/L)	3.2 ± 1.0	2.8 ± 0.9	2.3 ± 0.8	0.4 (0.1, 0.8)*	0.8 (0.5, 1.2)**
Apolipoprotein A (g/L)	1.4 ± 0.3	1.4 ± 0.2	1.5 ± 0.3	0.0 (-0.2, 0.2)	−0.1 (-0.3, 0.2)
Apolipoprotein B (g/L)	1.1 ± 0.4	1.1 ± 0.1	1.4 ± 0.5	0.0 (-0.2, 0.2)	−0.3 (-0.6, 0.0)
Inflammatory factors					
IL-6	26.7 ± 12.8	17.6 ± 3.0	19.7 ± 4.0	9.1 (1.6, 16.6)*	7.0 (−1.2, 15.2)
IL-8	14.8 (11.3, 17.9)	11.6 (11.4, 13.9)	12.1 (11.3, 12.6)	9.2 (-6.4, 24.8)	9.7 (-6.0, 25.3)
MCP-1	192.0 ± 130.8	156.1 ± 65.6	142.6 ± 91.0	35.9 (-14.5, 86.4)	49.4 (-1.6, 97.3)*
IL-18	68.8 (20.9,186.1)	27.1 (18.1,122.9)	34.5 (17.0,108.5)	30.4 (-4.0, 64.9)	18.5 (28.3, 65.3)
IL-37	33.3 ± 16.0	29.0 ± 8.0	28.0 ± 5.4	4.3 (-2.2, 10.8)	5.4 (-2.9, 13.7)
TNF-α	34.5 ± 26.4	25.4 ± 5.0	25.4 ± 3.8	9.2 (-6.3, 24.6)	9.1 (-8.4, 26.6)
Endothelial Cell Function measurements					
sICAM-1	4.8 × 10^5^ ± 2.3 × 10^5^	4.1 × 10^5^ ± 1.3 × 10^5^	4.2 × 10^5^ ± 1.65 × 10^5^	0.7 × 10^5^ (-0.7 × 10^5^, 2.2 × 10^5^)	0.5 × 10^5^ (-0.7 × 10^5^, 1.7 × 10^5^)
VCAM-1	6.8 × 10^5^ ± 3.2 × 10^5^	4.7 × 10^5^ ± 2.5 × 10^5^	5.3 × 10^5^ ± 3.95 × 10^5^	2.1 × 10^5^ (-1.0 × 10^5^, 5.2 × 10^5^)	1.5 × 10^5^ (1.8 × 10^5^, 4.8 × 10^5^)
P-selectin	1.1 × 10^5^ ± 0.6 × 10^5^	0.7 × 10^5^ ± 0.2 × 10^5^	0.8 × 10^5^ ± 0.35 × 10^5^	0.4 × 10^5^ (0.1 × 10^5^, 0.8 × 10^5^)*	0.4 × 10^5^ (0.01 × 10^5^, 0.7 × 10^5^)*
NO	250.8 ± 152.5	218.2 ± 153.8	158.5 ± 139.3	32.5 (-93.4,158.5)	92.3 (-33.6,218.2)
PGI2	99.5 ± 19.1	106.7 ± 23.0	115.5 ± 28.1	−7.1 (-10.9,-3.4)**	−15.9 (-23.9,-8.0)**
ET-1	96.5 ± 20.3	91.8 ± 19.8	84.8 ± 17.7	4.7 (2.9, 6.5)**	11.7 (9.3, 14.0)**
TF	56.7 ± 6.5	52.8 ± 6.0	49.4 ± 5.2	3.9 (2.6, 5.2)**	7.2 (5.9, 8.6)**
t-PA	16.7 ± 2.0	17.9 ± 1.9	18, 9 ± 2.4	−1.2 (-1.6,-0.8)**	−2.2 (-2.9,-1.5)**
vWF	192.5 ± 16.0	183.0 ± 14.9	175.8 ± 14.5	9.6 (7.2, 12.0)**	16.8 (14.7, 18.9)**
PAI-1	61.2 ± 6.4	57.6 ± 6.5	54.5 ± 6.1	3.6 (2.6, 4.5)**	6.7 (5.7, 7.7) **
Liver function-related measurements					
AST (U/L)	22.0 (20.8, 28.3)	21.1 (17.2, 24.4)	21.4 (116.9, 27.5)	25.8 (-20.7, 72.4)	25.1 (-21.4, 71.7)
ALT (U/L)	27.8 (22.5, 71.6)	25.7 (21.7, 34.6)	24.0 (16.6, 32.7)	13.8 (-2.5, 30.1)	17.4 (-1.3, 36.0)
GGT (U/L)	31.7 (24.7,204.7)	28.1 (21.8,189.2)	28.8 (22.9,103.3)	101.0 (-68.6,270.6)	126.7 (-55.8,309.3)
ALP (U/L)	67.5 (60.0,121.8)	64.0 (52.0, 80.3)	62.5 (52.5, 72.8)	23.6 (-3.0, 50.1)	31.8 (-2.8, 66.3)
TBIL (μmol/L)	10.5 ± 4.0	9.6 ± 2.3	9.3 ± 2.7	0.9 (-1.5, 3.3)	1.3 (-1.2, 3.7)
DBIL (μmol/L)	3.7 ± 1.6	3.2 ± 1.0	3.3 ± 1.0	0.5 (-0.3, 1.4)	0.4 (-0.4, 1.3)
Renal function-related measurements					
UA (μmol/L)	312.8 ± 142.2	326.9 ± 182.3	353.9 ± 172.2	−14.2 (-61.9, 33.6)	−41.2 (-88.9, 6.6)
BUN (mmol/L)	6.1 ± 3.0	7.0 ± 2.5	6.4 ± 2.4	−0.9 (-2.8, 1.0)	0.3 (-1.7, 1.1)
Scr (μmol/L)	67.6 ± 38.0	74.9 ± 39.6	72.0 ± 42.7	−7.3 (-14.5,-0.2)*	−4.4 (-13.5, 4.7)
Cystatin (mg/L)	0.9 ± 0.4	0.9 ± 0.4	0.9 ± 0.4	0.0 (-0.1.0.1)	0.0 (-0.1.0.1)

Note: BMI, body mass index; WC, waist circumference; HbA1c, glycated hemoglobin; FBG, fasting blood glucose; 2HPG, 2-h postprandial blood glucose; FCP, fasting c-peptide; 2HCP, 2-h postprandial c-peptide; FINS, fasting insulin; 2INS: 2-h postprandial insulin; HOMA-IR, homeostasis model assessment of insulin resistance; HOMA-β, HOMA-β, cell function index; TG, total triglycerides; TC, total cholesterol; LDLC, low-density lipoprotein cholesterol; HDLC, high-density lipoprotein cholesterol; IL-6, interleukin-6; TNF-α, tumor necrosis factor α. MCP-1, monocyte chemoattractant protein-1; sICAM-1, Soluble cell adhesion molecules-1; VCAM-1, Vascular Cell Adhesion Molecule-1; NO, nitric oxide; PGI2, prostacyclin; ET-1, Endothelin-1; t-PA, tissue type fibrinogen activator; vWF, von willebrand factor; PAI-1, plasminogen activator inhibitor-1 ALT, alanine aminotransferase; AST, aspartate aminotransferase; ALP, alkaline phosphatase; GGT, γ-glutamyl transpeptidase; TBIL, total bilirubin; DBIL, direct bilirubin; UA, uric acid; BUN, urea nitrogen; Scr, serum creatinine. Data are presented as the mean ± SD.*P < 0.05; **P < 0.001.

### 3.2 SGLT2i alters the composition of the gut microbiota in T2DM patients

**FIGURE 2:**
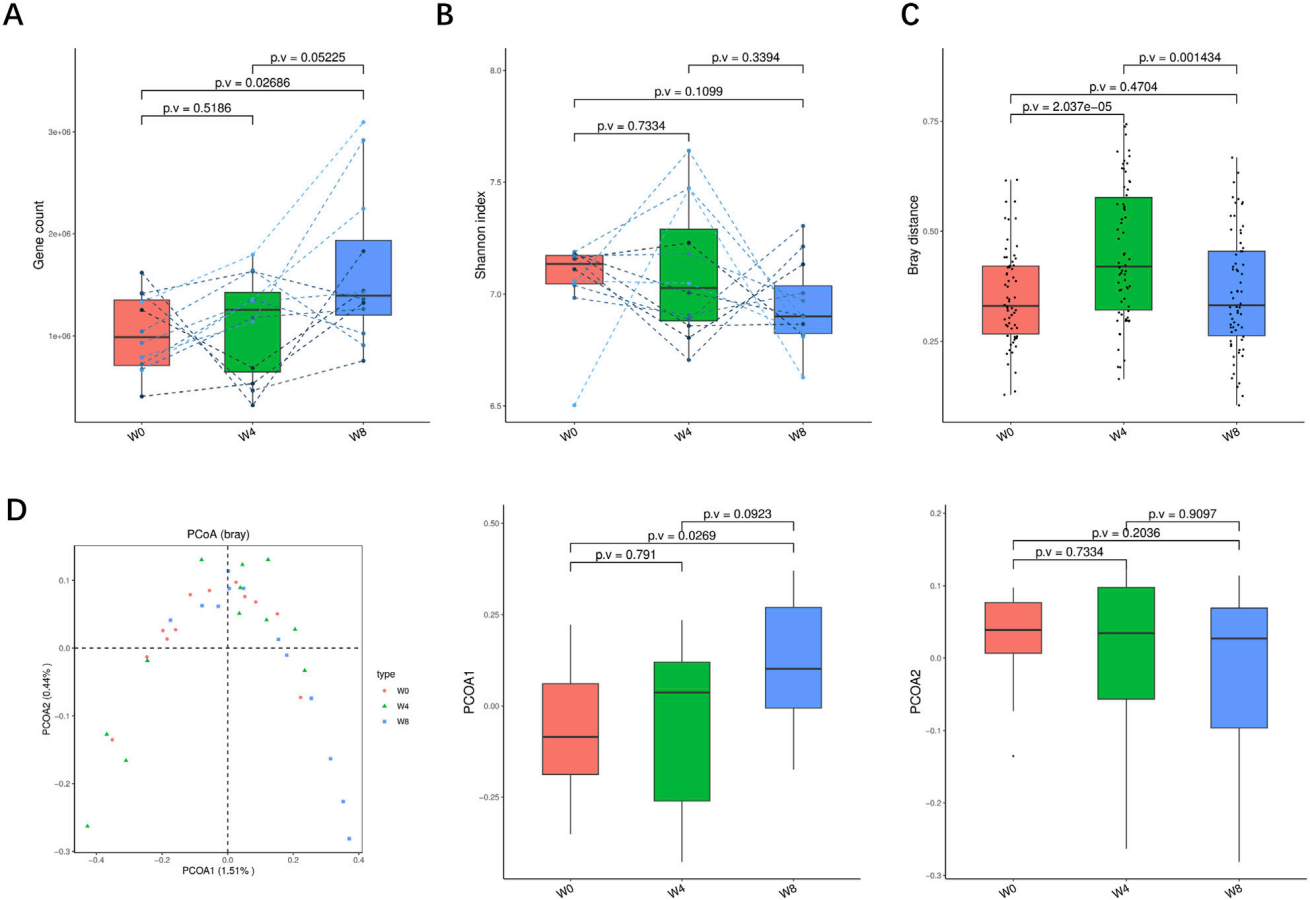
Alpha-diversity, beta-diversity, and principal component analysis at the gene level. **(A)** Gene count in comparison between W0 and W4, W0 and W8, and W4 and W8. **(B)** Shannon index in comparisons between W0 and W4, W0 and W8, and W4 and W8 **(C)** Beta diversity based on Bray-Curtis (Bray) distance for W0 vs. W4, W0 vs. W8, and W4 vs. W8. **(D)** PCoA analysis based on Bray distance showed that the first and second principal components were detected in W0 vs. W4, W0 vs. W8, and W4 vs. W8.

**FIGURE 3:**
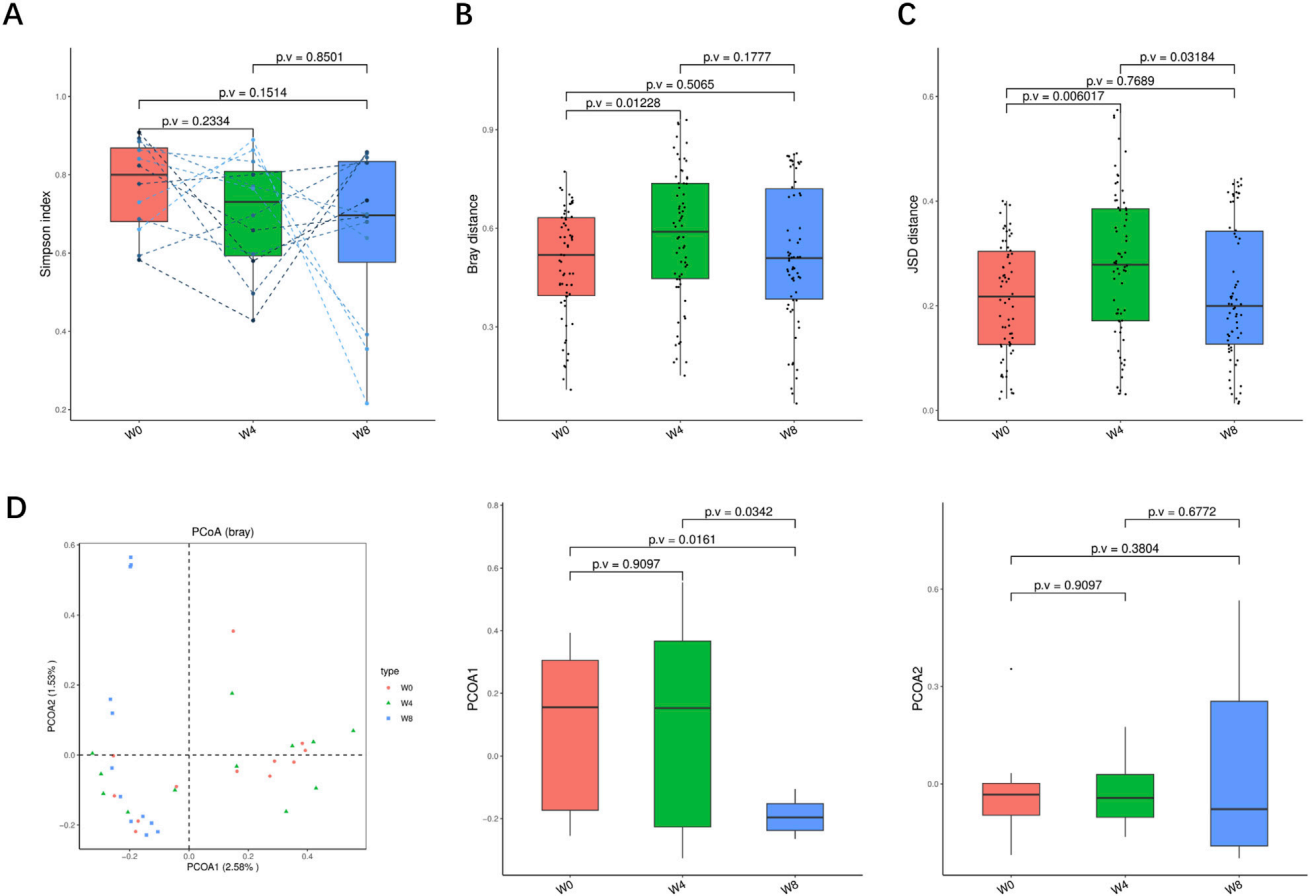
Alpha-diversity, beta-diversity, and principal component analysis at the genus level. **(A)** Simpson index in comparison between W0 and W4, W0 and W8, and W4 and W8. **(B)** Beta diversity based on Bray-Curtis (Bray) distance for W0 vs. W4, W0 vs. W8, and W4 vs. W8. **(C)** The Jensen-Shannon Divergence (JSD) distance-based beta diversity in W0 vs. W4, W0 vs. W8, and W4 vs. W8. **(D)** PCoA analysis based on Bray distance showed that the first and second principal component was detected in W0 vs. W4, W0 vs. W8, and W4 vs. W8.

**FIGURE 4:**
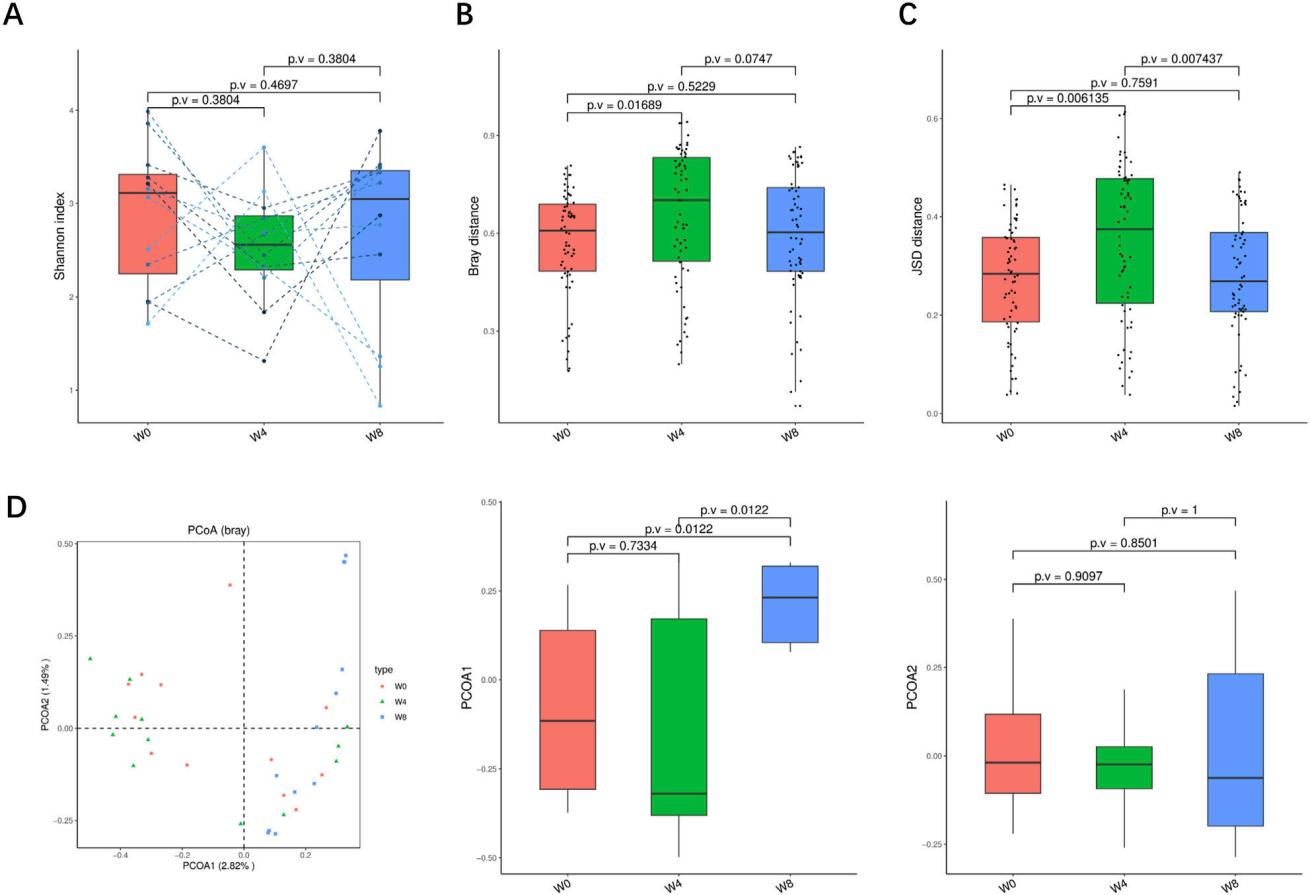
Alpha-diversity, beta-diversity, and principal component analysis at the species level. **(A)** Shannon index in comparison between W0 and W4, W0 and W8, and W4 and W8. **(B)** Beta diversity based on Bray-Curtis (Bray) distance for W0 vs. W4, W0 vs. W8, and W4 vs. W8. **(C)** The Jensen-Shannon Divergence (JSD) distance-based beta diversity in W0 vs. W4, W0 vs. W8, and W4 vs. W8. **(D)** PCoA analysis based on Bray distance showed that the first and second principal component was detected in W0 vs. W4, W0 vs. W8, and W4 vs. W8.

**FIGURE 5:**
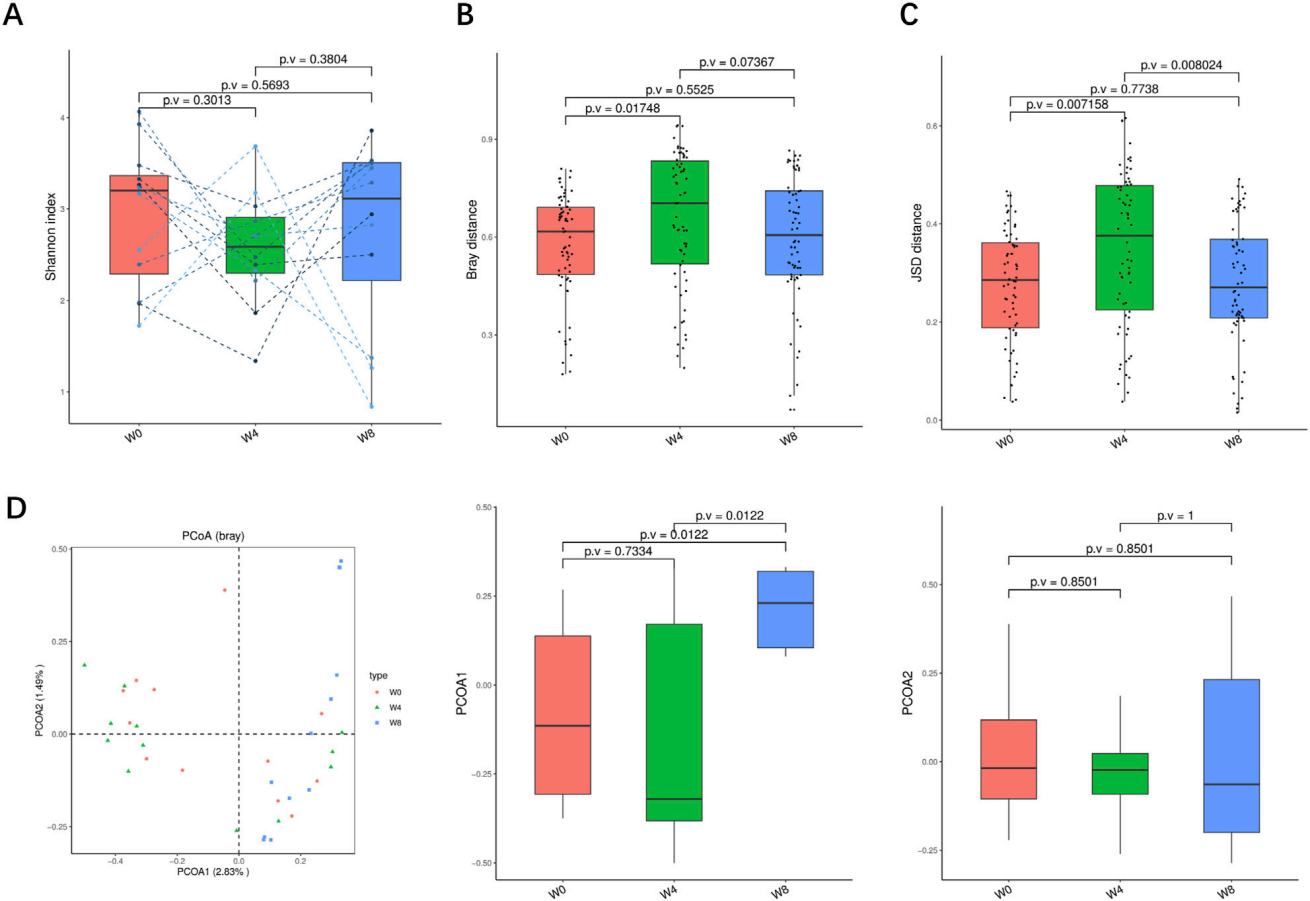
Alpha-diversity, beta-diversity, and principal component analysis at the strain level. **(A)** Shannon index in comparison between W0 and W4, W0 and W8, and W4 and W8. **(B)** Beta diversity based on Bray-Curtis (Bray) distance for W0 vs. W4, W0 vs. W8, and W4 vs. W8. **(C)** The Jensen-Shannon Divergence (JSD) distance-based beta diversity in W0 vs. W4, W0 vs. W8, and W4 vs. W8. **(D)** PCoA analysis based on Bray distance showed that the first and second principal component was detected in W0 vs. W4, W0 vs. W8, and W4 vs. W8.

A total of 36 stool samples from 12 T2DM patients were analyzed at W0, W4, and W8 treatment with SGLT2i. Diversity analysis at the gene levels, including gene stripes (A), Shannon index (B), Bray index (C) and principal component analysis (D) were performed as shown in Figure 2. The numbers of genes showed an increasing trend after 4 weeks (*p* > 0.05) and 8 weeks of SGLT2i use (*p* < 0.05). Shannon index showed a decreasing trend after SGLT2i treatment (*p* > 0.05). Bray index showed an increasing trend at 4 weeks (*p* < 0.01) and then recovered at 8 weeks after SGLT2i treatment (*p* > 0.05). The Principal Coordinate Analysis (PCoA) indicated that the gut microbiome of the patients showed a significant shift at 8 weeks after SGLT2i treatment (*p* < 0.05). There were similar trends of changes at the genus, species and strain levels (Figures 3–5).

**FIGURE 6:**
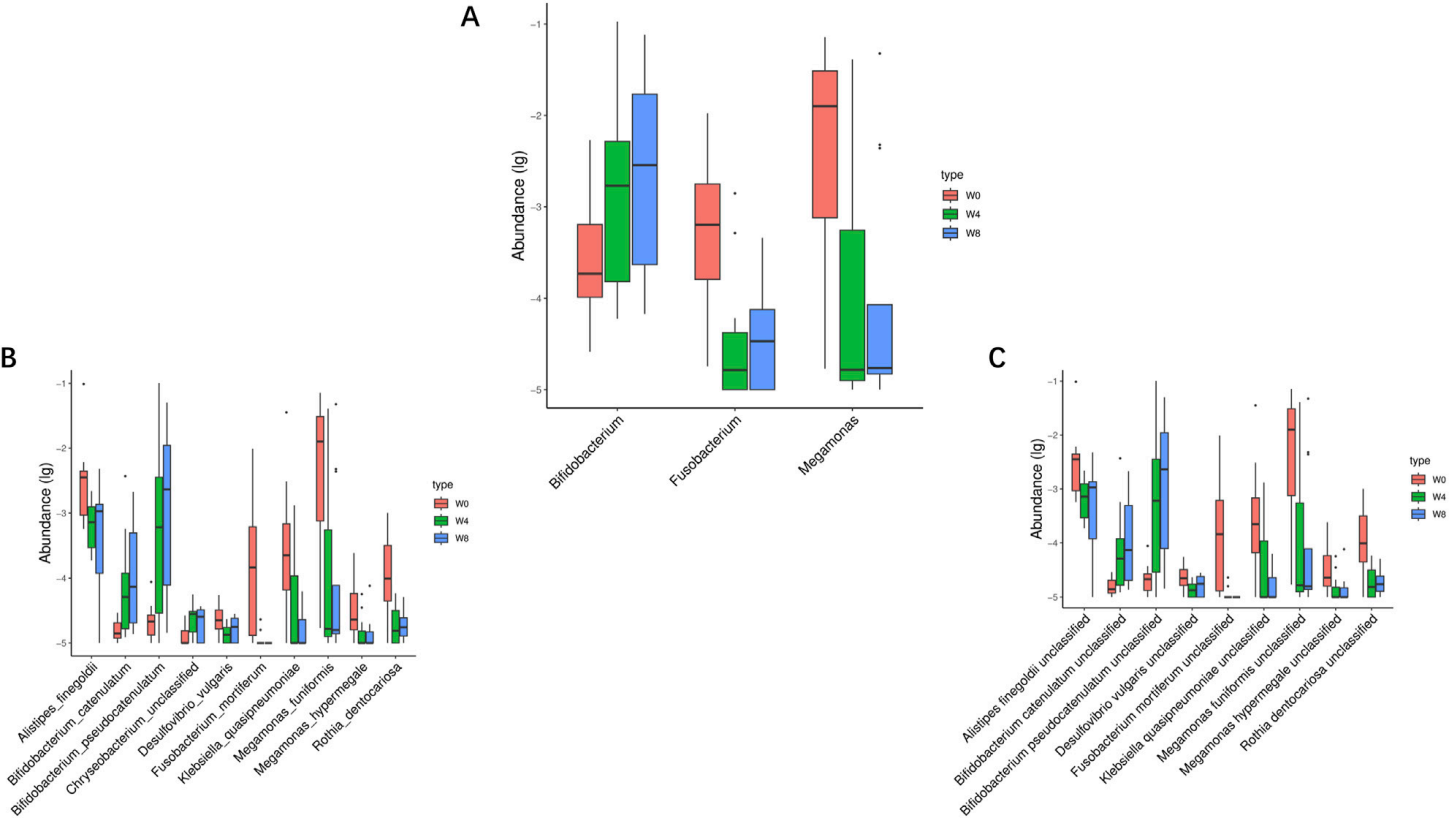
Significantly different taxa at levels of genus **(A)**, species **(B)** and strain **(C)** in comparison between W0 vs. W4, W0 vs. W8, and W4 vs. W8.

Next, we analyzed the significant differences of intestinal microbiota in T2DM patients treated with SGLT2i. At the genus level, three types of bacteria were significantly changed: *Bifidobacterium*, *Fusobacterium*, *Megamonas*. After SGLT2i treatment, an increasing trend of *Bifidobacterium* and decreasing trends of *Fusobacterium*, *Megamonas were* observed (Figure 6A). At the species level, 10 types of bacteria were significantly changed: *Alistipes_finegoldii*, *Bifidobacterium catenulatum*, *Bifidobacterium_ pseudocatenulatum*, *Chryseobacterium_unclassified*, *Desulfovibrio vulgaris*, *Fusobacterium_mortiferum*, *Klebsiella_quasipneumoniae*, *Megamonas_funiformis*, *Megamonas_hypermegale* and *Rothia_dentocariosa* (Figure 6B). At the strain level, nine types of bacteria were significantly changed: *Alistipes finegoldii unclassified*, *Bifidobacterium catenulatum unclassified*, *Bifidobacterium pseudocatenulatum unclassified*, *Desulfovibrio vulgaris unclassified*, *Fusobacterium mortiferum unclassified*, *Klebsiella quasipneumoniae unclassified*, *Megamonas funiformis unclassified*, *Megamonas hypermegale unclassified* and *Rothiadentocariosa unclassified* (Figure 6C). These results suggested that SGLT2i significantly altered the structure and composition of intestinal microflora in T2DM patients.

### 3.3 SGLT2i changes the functional pathways of the gut microbiota in T2DM patients

**FIGURE 7:**
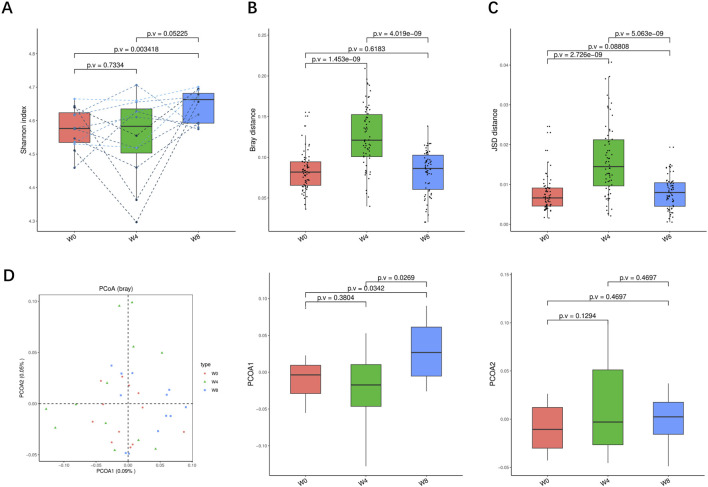
Alpha-diversity, beta-diversity, and principal component analysis at the KO level. **(A)** Shannon index in comparison between W0 and W4, W0 and W8, and W4 and W8. **(B)** Beta diversity based on Bray-Curtis (Bray) distance for W0 vs. W4, W0 vs. W8, and W4 vs. W8. **(C)** The Jensen-Shannon Divergence (JSD) distance-based beta diversity in W0 vs. W4, W0 vs. W8, and W4 vs. W8. **(D)** PCoA analysis based on Bray distance showed that the first and second principal component was detected in W0 vs. W4, W0 vs. W8, and W4 vs. W8.

To verify the change of functional pathways, KO—Alpha Diversity, KO—beta Diversity, KO—Principal Coordinate Analysis (Bray) were analyzed and found that there were changes in the functions of bacteria with signals, modules, and pathways (Figure 7). Shannon index showed an increasing trend after SGLT2i treatment at 4 weeks (*p* > 0.05) and 8 weeks (*p* < 0.01). Bray distance showed an increasing trend after SGLT2i treatment at 4 weeks (*p* < 0.01) and then recovered at 8 weeks (*p* < 0.01). JSD distance showed a similar trend (*p* < 0.05). The PCoA analysis indicated a significant shift of gut microbiome in T2DM patients at 8 weeks after SGLT2i treatment (*p* < 0.05).

### 3.4 Cardiovascular benefits of SGLT2i associated with gut microbiota

**FIGURE 8:**
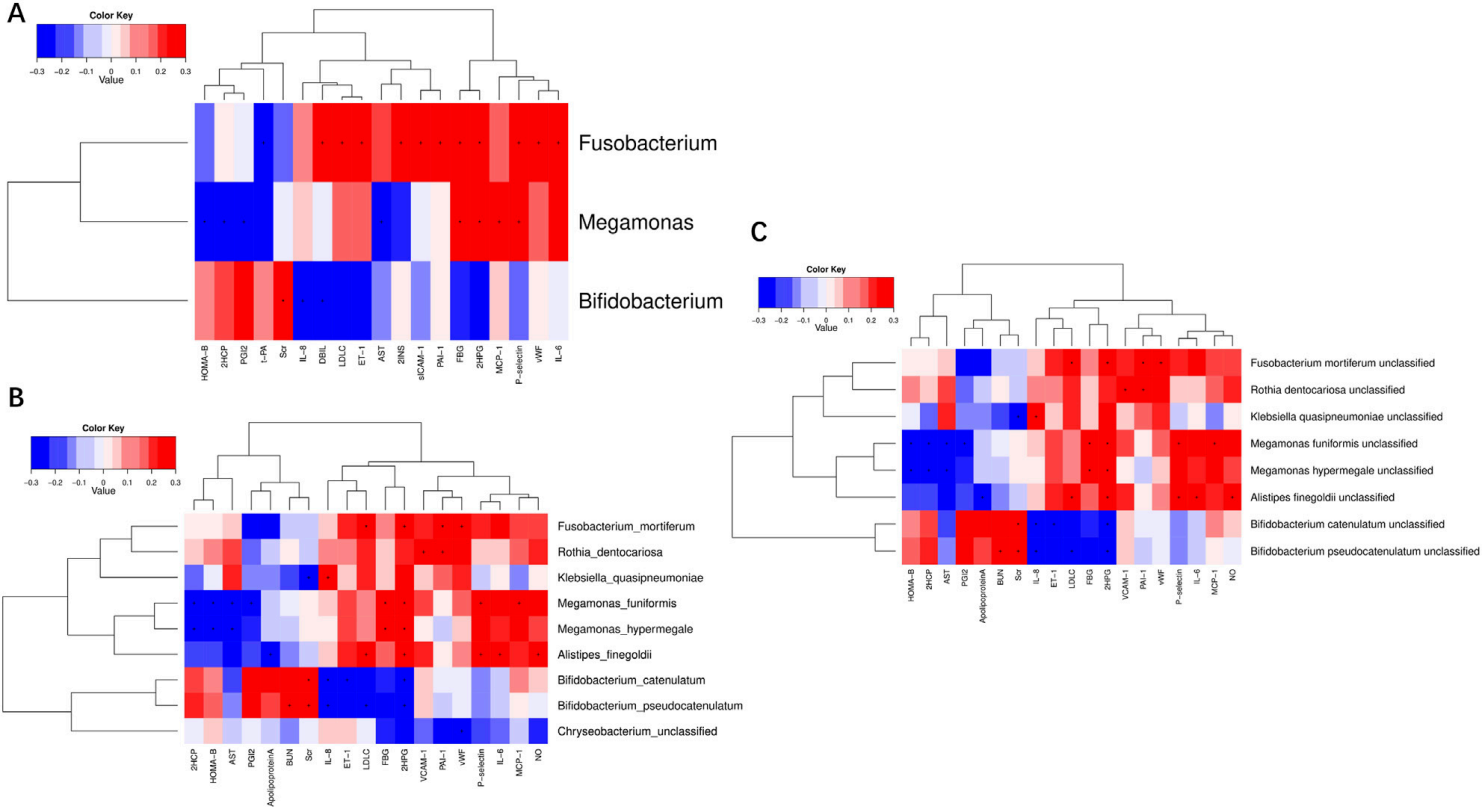
Correlation analysis of significantly different clinical indicators and differential bacteria at levels of genus **(A)**, species **(B)** and strain **(C)** in type 2 diabetic patients.

Correlation analysis was undertaken between the alterations in gut microbiota and the clinical indicators in T2DM patients treated with SGLT2i. At the genus level, *Fusobacterium* was positively correlated with 2INS, 2HPG, FBG, LDLC, DBIL, IL-6, P−selectin, ET−1, vWF, sICAM-1, PAI-1, and negatively correlated with t−PA. *Megamonas* was positive correlated with 2HPG, FBG, MCP-1, P−selectin and negatively correlated with AST, 2HCP, HOMA-β, PGI2. *Bifidobacterium* was positively correlated with Scr and negatively correlated with IL-8, DBIL (Figure 8A). At the species level, *Fusobacterium_mortiferum* was positively correlated with 2HPG, LDLC, PAI−1, vWF. *Megamonas_funiformis* was positively correlated with 2HPG, FBG, P−selectin, MCP-1 and negatively correlated with AST, 2HCP, HOMA-β, PGI2. *Megamonas_hypermegale* was positively correlated with 2HPG, FBG and negatively correlated with AST, 2HCP, HOMA-β. *Bifidobacterium_catenulatum* was positively correlated with Scr and negatively correlated with IL-8, 2HPG, ET-1. *Bifidobacterium_ pseudocatenulatum* was positively correlated with BUN, Scr and negatively correlated with IL-8, LDLC (Figure 8B). At the strain level, *Fusobacterium mortiferum unclassified* was positively correlated with 2HPG, LDLC, PAI−1 and vWF. *Megamonas funiformis unclassified* was positively correlated with 2HPG, FBG, MCP-1, P−selectin and negatively correlated with AST, 2HCP, HOMA-β, PGI2. *Megamonas hypermegale unclassified* was positively correlated with 2HPG, FBG and negatively correlated with AST, 2HCP, HOMA-β. *Bifidobacterium catenulatum unclassified* was positively correlated with Scr and negatively correlated with IL-8, 2HPG, ET-1. *Bifidobacterium pseudocatenulatum unclassified* was positively correlated with BUN, Scr and negatively correlated with IL-8, LDLC, 2HPG (Figure 8C). These results suggest that the cardiovascular benefits of SGLT2i were related to the alteration of intestinal microflora in T2DM patients.

## 4 Discussion

**FIGURE 9:**
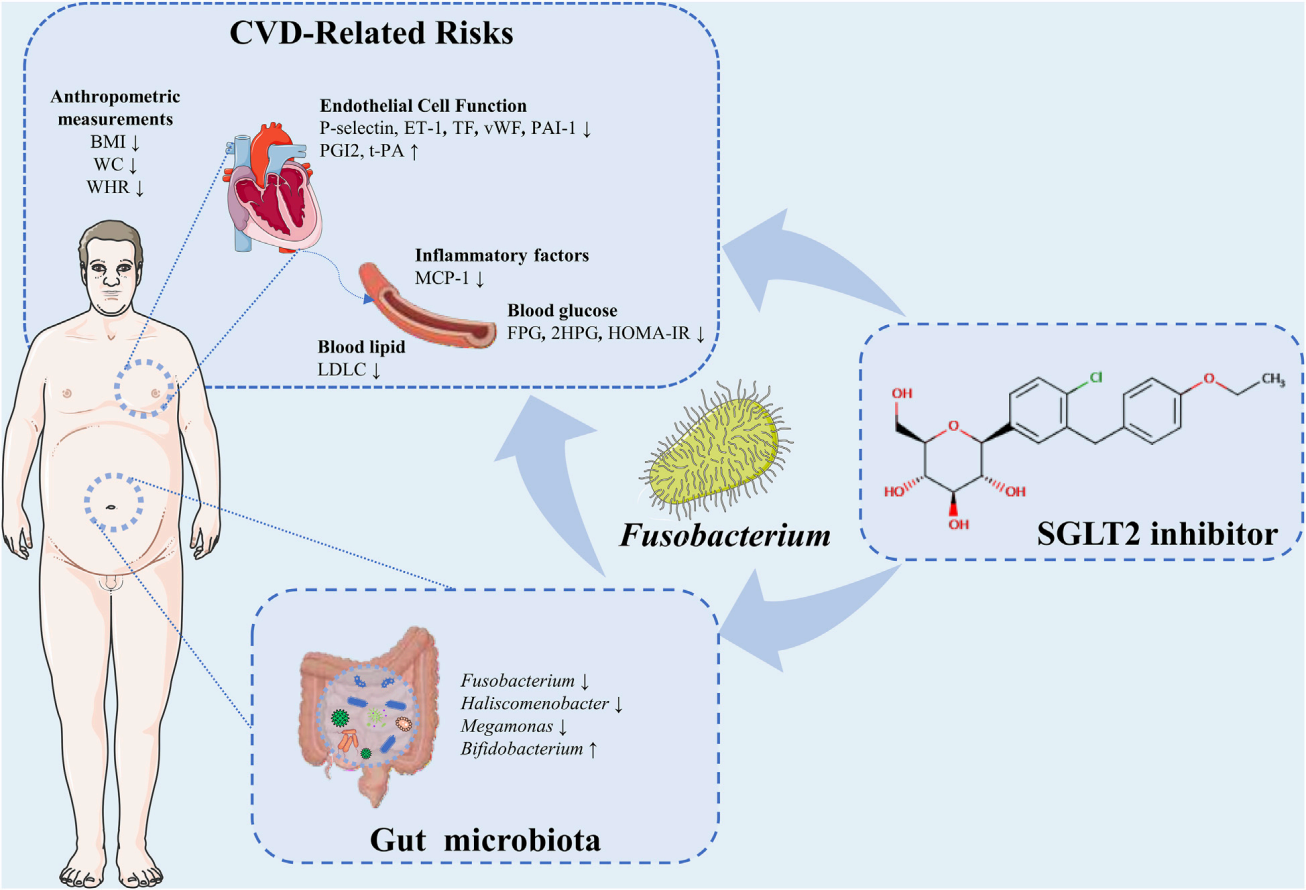
A schema summarizing the findings of this study.

In the present study, we verified the cardiovascular benefits and alteration of gut microbiota in T2DM patients with SGLT2i treatment. We found that the improvement of risk factors for CVDs was associated with gut microbiota, particularly, the reduced abundance of *Fusobacterium* (Figure 9).

The DECLAR-TIMI study reported that SGLT2i dapagliflozin reduced the risk of major adverse cardiovascular events in patients with T2DM and a history of atherosclerotic cardiovascular disease (Furtado et al., 2019; Perrone-Filardi et al., 2017). In the present study, the improvements were observed in CVD-related indicators in the patients, encompassing body weight parameters, glucose-related parameters, blood lipid-related parameters, inflammatory factors, and endothelial cell function-related parameters following SGLT2i treatment. In addition to obesity, hyperglycemia, dyslipidemia and hypertension, which are the most common CVD risk factors (Marx et al., 2023), endothelial dysfunction is an independent predictor of CVDs in patients with T2DM (de Jager et al., 2006). Recently, several trials have demonstrated the efficacy of dapagliflozin in improving endothelial function in patients with T2DM (Shigiyama et al., 2017; Sposito et al., 2021). Increased P-selectin levels were positively associated with the risk of CVDs in T2DM groups (Alzahrani et al., 2023). There was clinical evidence for elevated plasma ET-1 levels in patients with T2DM, leading to endothelial dysfunction, which are actively involved in the pathophysiology of the onset and progression of coronary artery disease (Zhang et al., 2020; Yang et al., 2024). High vWF levels had predictive values for screening patients with T2DM accompanied by endothelial dysfunction (Li et al., 2023). Hypo-fibrinolysis is a key character in diabetes and contributes to CVD in patients. The increased concentration of PAI-1 impedes fibrinolysis by impairing the action of t-PA, therefore thrombi cannot be removed from the vascular wall (Altalhi et al., 2021). PGI2 is involved in vasodilation, anti-inflammatory and Anti-thrombosis (Majed and Khalil, 2012). Endothelial dysfunction in patients suffering from T2DM is associated with suppressed release of PGI2 (Onat et al., 2011). As the CVD continuum is triggered by a multitude of risk factors, ultimately leading to the development of end-stage CVD (Dzau et al., 2006), SGLT2i treatment may help delay the progression of CVD by managing these risk factors.

Emerging evidence showed that the gut microbiota was involved in chronic inflammation, metabolic disorders and oxidative in the host, contributing to the progression of T2DM and CVDs (Yang et al., 2021; Wu et al., 2023; Nemet et al., 2020; Wang et al., 2011; Raygan et al., 2018; Yuan et al., 2019). A randomized, double-blind, placebo-controlled trial reported that probiotic supplementation significantly decreased fasting plasma glucose, insulin resistance, serum high sensitivity C-reactive protein, and led to a significant elevation in total antioxidant capacity in T2DM patients with coronary heart disease (Raygan et al., 2018). Diabetes mellitus and atherosclerosis are associated with some similar alterations in the composition of the gut microbiota composition and its metabolites, such as increased lipopolysaccharides (LPS), trimethylamine N-oxide and phenylacetylglutamine. In mechanism level, LPS produced by gram-negative bacteria might lead to metabolic endotoxemia and LPS -dependent production of inflammatory cytokines, such as Interleukin-6 and Tumour Necrosis Factor-α that can contribute to development of insulin resistance and T2DM (Duan et al., 2018; Schwartz et al., 2016). In metabolism-dependent pathways, the altered gut microbiota and some of their metabolites would affect lipid metabolism and contribute to elevated triglycerides and decreased high-density lipoprotein in CVD patients (Fu et al., 2015). A functional study demonstrated that direct provision of T trimethylamine N-oxide, a gut microbiota metabolites, accelerated atherosclerosis in murine models, and that suppression of gut microbiota dependent conversion of nutrient precursors (choline) into trimethylamine N-oxide blocked choline diet dependent enhancement in atherosclerosis (Wang et al., 2011). Another gut microbiota-derived metabolite, phenylacetylglutamine was reported to be associated with CVD acts via adrenergic receptors in both diabetics and non-diabetics (Nemet et al., 2020). Besides, increased abundance of *Escherichia coli* could elevate production of uric acid, which contributes to the overproduction of oxygen free radicals, vascular endothelial dysfunction, and inflammation (Chistiakov et al., 2015; Kang and Ha, 2014). Herein, SGLT2i changed the structure and function of intestinal flora in T2DM patients. Dustinet et al. (Lee et al., 2018) reported that dapagliflozin improved generalized vascular dysfunction associated with gut microbiota alteration in type 2 diabetic mice. Deng X. et al. (2022) proposed that the cardiovascular benefits of empagliflozin, another SGLT2 inhibitor, were associated with gut microbiota. However, another research showed that dapagliflozin could not altered the fecal microbiome in patients with T2DM (van Bommel et al., 2020). The inconsistency may be attributable to the unparallel analysis of gut microbiota and selection of the ethnicity, environment, diet and socio-economic variables (Ahmad et al., 2019).

In this study, SGLT2i increased relative abundance of the probiotic *Bifidobacterium* and decreased relative abundances of harmful bacteria, including *Fusobacterium* and *Megamonas*. The results of published studies differ, but in general, the genera negatively associated with T2DM are *Bifidobacterium*, *Bacteroides, Akkermansia* and *Roseburia,* while the genera *Fusobacteria*, *Firmicutes* and *Ruminococcus* were positively associated with T2DM (Metwaly et al., 2022; Tanase et al., 2020). Probiotics *Bifidobacterium* was considered to improve metabolic endotoxemia and glucose intolerance (Reitmeier et al., 2020). *Bifidobacterium* ferments to produce short-chain fatty acids, which have many health-promoting properties, including the maintenance of intestinal barrier integrity and anti-inflammatory function (Deng K. et al., 2022). It was reported that dapagliflozin increased the abundance of *Bifidobacterium*in in a T2DM rat model (Yang M. et al., 2020). In addition, *Megamonas* was associated with higher levels of HbA1c in T2DM (Ren et al., 2023). *Fusobacterium* is a highly adhesive and invasive phylum, which colonize at host mucosa and secrete a variety of toxins and enzymes (Gurung et al., 2020), and was considered as a pathogenic anaerobic organism in T2DM patients (Tanase et al., 2020; Metwaly et al., 2022).

To the best of our knowledge, this is the first study to reported that SGLT2i reduced the abundance of *Fusobacterium*. Surprisingly, *Fusobacterium* was positively correlated with 2INS, 2HPG, FBG, LDLC, DBIL, IL-6, P−selectin, ET−1, vWF, sICAM-1, PAI-1, and negatively correlated with t−PA. These results suggested that the hypoglycemic, lipid reduction, anti-inflammatory, vascular endothelial protective effects of SGLT2i were associated with alteration of intestinal flora, particularly the reduced abundance of *Fusobacterium*. Consistence to our results, *Fusobacterium* was positively associated with glucose metabolic disorders and insulin resistance in diabetes and non-diabetes (He et al., 2024; Du et al., 2021). Recent research indicated that quercetin could reduce TC, TG, and LDLC levels in hyperlipidemia rat which associated with changed intestinal flora including *Fusobacterium* (Wang T. et al., 2023). Bile acid (BA) receptors are expressed in endothelial cells indicating the relevance of BAs to CVD. Hydrophobic BAs are cardiotoxic, while hydrophilic BAs are cardioprotective (Zhang et al., 2024). Recent study found that the downstream hydrophilic BA products (e.g., glycochenodeoxycholic acid, and glycoursodeoxycholic acid) of *Fusobacterium* might account for the correlation between *Fusobacterium* and endothelial dysfunction (Zhuo et al., 2024). *Fusobacterium_nucleatum*, a specific species of *Fusobacterium*, was consider to an potentially pathogenic bacteria in several disorders with endothelial damage (Farrugia et al., 2022) and atherosclerosis (Wang Z. et al., 2023). Cheng et al. (2018) reported that the enrichment of *Fusobacterium* corelated with low-grade inflammation at intestinal mucosal epithelium, and this inflammation might drive the microbial dysbiosis into a positive feedback loop with altered host response. Additionally, *Fusobacterium* might involved in activating the endoplasmic reticulum stress pathway to promote intestinal mucosal barrier destruction (Cheng et al., 2018). Either intestinal mucosal barrier damage (Teixeira et al., 2012; Cox et al., 2017; Ferrucci and Fabbri, 2018) or microbial dysbiosis (Amabebe et al., 2020; Qin et al., 2012; Yang et al., 2021; Zhao, 2013) related disorder of metabolism or inflammation have been reported in several diseases including obesity, type 2 diabetes and CVD. Overall, the correlation of *Fusobacterium* in diabetes and CVD was noticed by several studies, and the intervention effect of SGLT2 targeting *Fusobacterium* was indicated.

Nevertheless, our study has some limitations. Firstly, the study is a single-arm design. Secondly, the sample size was relatively small. In addition, the follow-up period was insufficient to evaluate the long-term effects of SGLT2i on cardiovascular events in T2DM patients. Lastly, the plasma proteomics and metabolites of patients requires further investigation.

In conclusion, the cardiovascular benefits of SGLT2i are not solely ascribed to its inhibition of SGLT2. Our works indicate that modulation of the gut microbiota, particularly *Fusobacterium*, may be one of the mechanisms involved.

## Data Availability

The raw sequencing data supporting the conclusions of this article were deposited in the NCBI Sequence Read Archive (SRA) database under bioproject number PRJNA1238196. (https://www.ncbi.nlm.nih.gov/sra/PRJNA1238196).
