# Supplementary material for: Reduced abundance of Fusobacterium signifies cardiovascular benefits of sodium glucose cotransporter 2 inhibitor in type 2 diabetes: a single arm clinical trial
Source: Front Pharmacol. 2025 May 8;16:1600464. doi: 10.3389/fphar.2025.1600464 (PMC12095364; doi:10.3389/fphar.2025.1600464)
Supplement: Supplementary file 1 [file Supplementaryfile1.docx]

Supplementary Material

# Supplementary Data

HbA1c was measured by H9 HbA1c Testing System (Lifotronic, Shenzhen, China). FINS, 2-hour 2INS, FCP, 2HCP were measured by E602 Electrochemiluminescence immunoassay analyzer (Roch, Basel, Switzerland). FBG, 2HPG, TC, TG, HDLC, LDLC, Apolipoprotein A, Apolipoprotein B, AST, ALT, GGT, ALP, TBIL, DBIL, GGT, TBIL, DBIL, UA, BUN, Scr, Cystatin were measured by AU5800 automatic biochemical analyser (beckman coulter, USA). HOMA-IR = (FPG × FINS) / 22.5. HOMA β=20×FINS/(FPG-3.5).

Plasma levels of cytokines were measured using standard kits (4A biotech, Suzhou, China or CUSABIO, Wuhan, China) on a Tecan Infinite M1000 Pro Microplate reader (Tecan, Männedorf, Switzerland). IL-6 (CHE0009), IL-8 (CHE0011), IL-18 (CHE0007), IL-37 (CHE0174), MCP-1 (CHE0103), TNF-α (CHE0019), sICAM-1 (CHE0052), VCAM-1 (CHE0110), P-selectin (CHE0106), NO (CSB-E08322h), PGI2(CSB-E09591h), ET-1 (CSB-E07007h), TF (CSB-E07913h), t-PA (CSB-E07916h), vWF (CSB-E08437h), PAI-1(CSB-E07946h).
